# High-density genetic linkage map construction by F2 populations and QTL analysis of early-maturity traits in upland cotton (*Gossypium hirsutum* L.)

**DOI:** 10.1371/journal.pone.0182918

**Published:** 2017-08-15

**Authors:** Libei Li, Shuqi Zhao, Junji Su, Shuli Fan, Chaoyou Pang, Hengling Wei, Hantao Wang, Lijiao Gu, Chi Zhang, Guoyuan Liu, Dingwei Yu, Qibao Liu, Xianlong Zhang, Shuxun Yu

**Affiliations:** 1 State Key Laboratory of Cotton Biology, Institute of Cotton Research of CAAS, Anyang, Henan, China; 2 National Key Laboratory of Crop Genetic Improvement, College of Plant Science and Technology, Huazhong Agricultural University, Wuhan, Hubei, China; 3 Huanggang Academy of Agricultural Sciences, Huanggang, Hubei, China; 4 College of Agronomy, Northwest A&F University, Yangling, China; USDA-ARS Southern Regional Research Center, UNITED STATES

## Abstract

Due to China’s rapidly increasing population, the total arable land area has dramatically decreased; as a consequence, the competition for farming land allocated for grain and cotton production has become fierce. Therefore, to overcome the existing contradiction between cotton grain and fiber production and the limited farming land, development of early-maturing cultivars is necessary. In this research, a high-density linkage map of upland cotton was constructed using genotyping by sequencing (GBS) to discover single nucleotide polymorphism (SNP) markers associated with early maturity in 170 F_2_ individuals derived from a cross between LU28 and ZHONG213. The high-density genetic map, which was composed of 3978 SNP markers across the 26 cotton chromosomes, spanned 2480 cM with an average genetic distance of 0.62 cM. Collinearity analysis showed that the genetic map was of high quality and accurate and agreed well with the *Gossypium hirsutum* reference genome. Based on this high-density linkage map, QTL analysis was performed on cotton early-maturity traits, including FT, FBP, WGP, NFFB, HNFFB and PH. A total 47 QTLs for the six traits were detected; each of these QTLs explained between 2.61% and 32.57% of the observed phenotypic variation. A major region controlling early-maturity traits in *Gossypium hirsutum* was identified for FT, FBP, WGP, NFFB and HNFFB on chromosome D03. QTL analyses revealed that phenotypic variation explained (PVE) ranged from 10.42% to 32.57%. Two potential candidate genes, *Gh_D03G0885* and *Gh_D03G0922*, were predicted in a stable QTL region and had higher expression levels in the early-maturity variety ZHONG213 than in the late-maturity variety LU28. However, further evidence is required for functional validation. This study could provide useful information for the dissection of early-maturity traits and guide valuable genetic loci for molecular-assisted selection (MAS) in cotton breeding.

## Introduction

Upland cotton (*Gossypium hirsutum* L. AADD, 2n = 52), the most widely planted economic crop and the leading source of natural fiber worldwide, accounts for 95% of global cotton production [1]. Early maturity is an important breeding target in cotton cultivars; it can increase multiple crop indexes, reduce disaster losses and ensure stable cotton production. Short-season cotton, also called early-maturity cotton, generally exhibits a dwarf, compact plant architecture; shorter height of the node of the first fruiting branch (HNFFB); and shorter whole growth period (WGP) than those of middle- to late-maturity cotton. In recent years, with the limited arable land in China, there has been a competition between grain and cotton fiber production, which limits crop productivity. To address this and to efficiently utilize the limited farming land throughout the crop growing season, development of early-maturity cultivars with high yields and good fiber quality traits, coupled with resistance to major diseases, is needed. Flowering time (FT), the period from the first flower blooming to the first boll opening (FBP), WGP, node of the first fruiting branch (NFFB), HNFFB and plant height (PH) are important early-maturity-related traits of cotton [2–5]. WGP represents the entire duration of growth and development and consists of two periods: FT and FBP. All six traits are quantitatively inherited in cotton.

In cotton, the first genetic linkage map was constructed in 1994 using restriction fragment length polymorphism (RFLP) molecular markers from an interspecific F_2_ population of *G*. *hirsutum* and *G*. *barbadense* that comprised 705 loci and 57 lines. Even though single sequence repeat (SSR) markers are the most popular molecular markers in genetic map construction because of their specificity and simplicity, SSR markers cannot reach enough resolution for fine quantitative trait locus (QTL) mapping or map-based cloning. With the advancement of genome sequencing in cotton [6–9], several SNP markers have been identified and further assayed for polymorphisms, depending on the high-throughput platform [3, 10–16]. Among the different types of molecular markers, single nucleotide polymorphism (SNP) markers are currently the first choice for constructing genetic maps due to their high rate of polymorphism and various high-throughput automated platforms, such as Infinium, next-generation sequencing (NGS) and GoldenGate [17]. By using the high-throughput sequencing technologies, several thousand markers in parallel on automated platforms could be produced that are suitable for assaying. However, the utility of these technologies in high-density map construction and identification of qualitative trait loci (QTLs) in cotton using genome-wide linkage analysis needs to be explored. Linkage mapping and association mapping are powerful methods for detecting genetic loci underlying quantitative traits. Over the last two decades, many different quantitative traits in cotton have been reported, including fiber quality traits, yield and yield component traits, disease resistance traits, and drought tolerance-related traits [18–25]. However, early-maturity traits in cotton have received little attention, and to date, few QTLs related to early-maturity traits have been identified [3–5, 26–29]. For example, Fan et al. identified several QTLs related to FT and WGP in an F_2_ population derived from an upland cotton intraspecific cross [5]. Li et al developed two F_2:3_ populations and detected 4 common QTLs for early-maturity traits, including WGP, HNFFB, yield percentage before frost (YPBF) and the period from flower bud emergence to flowering (BP) on chromosome D03 [4]. Jia et al. constructed a high-density genetic map containing 6,295 SNPs and 139 SSR markers with an average interval of 0.63 cM and anchored one stable early-maturity-related QTL that spanned a 2-Mb region in 4 environments [10]. Su et al. employed association mapping techniques, which are different from bi-parental linkage mapping, using 81,675 high-density SNP markers in a set of 185 upland cotton accessions and identified 11 highly favorable SNP alleles for five early-maturity traits [3]. These results obtained through the study of early maturity in cotton may be valuable for improving cotton MAS breeding programs.

To better understand the genetic architecture of early-maturity traits in cotton, we present a linkage map generated from 3,978 SNPs that were developed through genotyping by sequencing (GBS). One major QTL was identified and explained 10.42–32.57% of the phenotypic variation (PV) in FT, FBP, WGP, NFFB and HNFFB. In addition, this QTL was also delimited to a 3.36-Mb interval on chromosome D03, a region spanning 112 genes. Candidate genes with known functions or *Arabidopsis* orthologs are also proposed. This study enriches our knowledge of the genetic bases of FT, FBP, WGP, NFFB, HNFFB and PH in cotton and provides valuable information for MAS breeding in the future.

## Materials and methods

### Parents and mapping population

**Fig 1:**
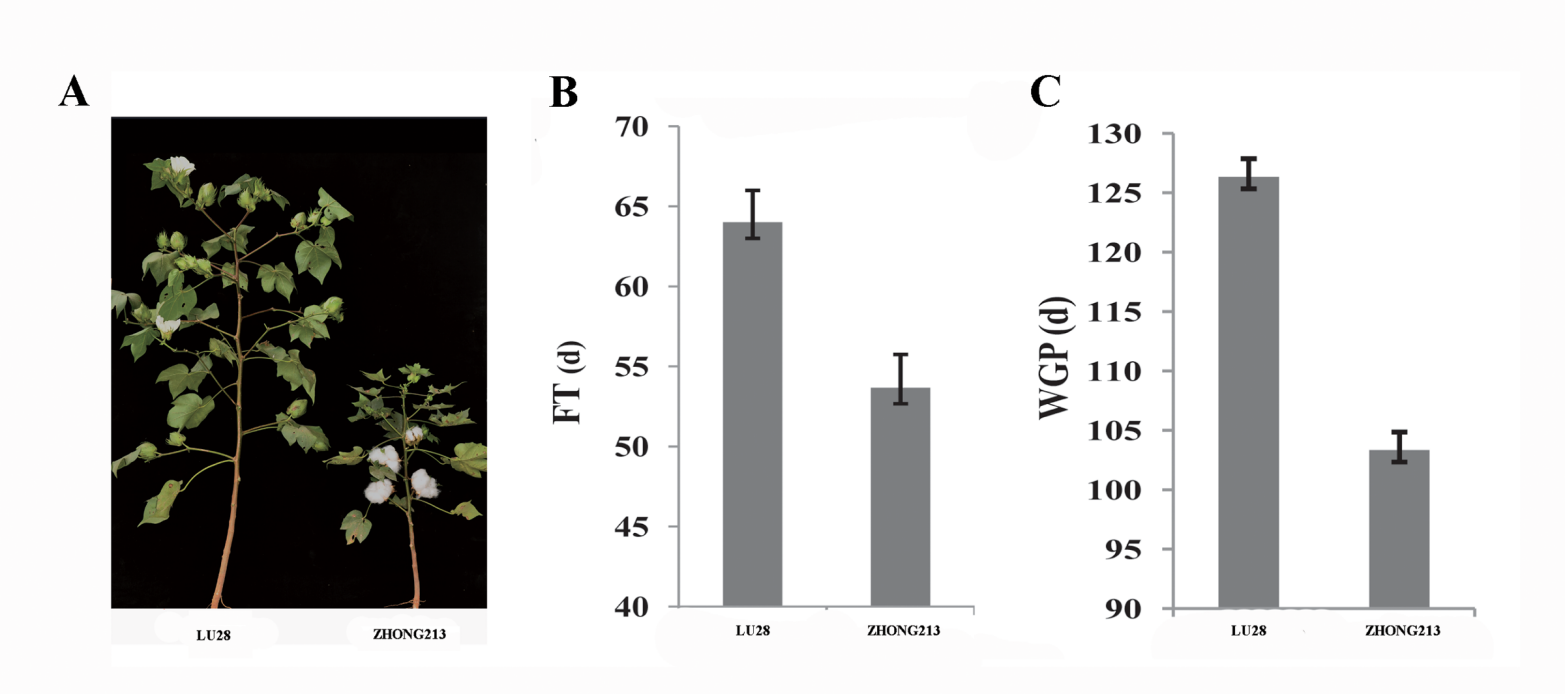
Flowering time (FT) and whole growth period (WGP) performance of two parents. **A** Phenotypes of LU28 and ZHONG213 with different FT and WGP. **B** Phenotypic effect values of FT for two parents. **C** Phenotypic effect values of WGP for two parents.

ZHONG213 and LU28 were used as parents. ZHONG213, a short-season upland cotton variety, was developed by the Cotton Research Institute of Chinese Academy of Agricultural Sciences (CRICAAS) from the cross (Mei-R1 × CRI27) × (Mei-R1 × Texas29-047). It is an excellent early-maturing cultivar and harbors strong early-maturity genes. ZHONG213’s pedigree was summarized in S1 Fig. In contrast, upland cotton strain LU28, a Chinese commercial multiple-hybrid line, was bred at the Shandong Cotton Research Center and exhibits late-maturity phenotypic traits and wider adaptability (Fig 1). In the summer of 2013, ZHONG213 was crossed with LU28 to obtain F_1_ seeds at CRICAAS, Anyang, Henan, China (36°08′N, 114°48′E). The F_1_ seeds were planted during winter in Sanya, Hainan, China (18°29′N, 109°52′E), and self-pollinated to produce F_2_ generation. The F_2_ seeds were planted in 10 rows (each 8 m long and 0.8 m apart) and self-pollinated to produce F_2:3_ seeds at Anyang in 2014. In 2015, we selected 170 F_2:3_ families and planted them in Anyang, Henan, China. The 170 F_2:3_ plants were grown in single-row plots with the same row length and width. Pesticides were used to control insects and diseases. The parental accessions used in the cross were obtained from the CRICAAS.

### Field experiments and phenotyping

The F_2_ and the F_2:3_ families were planted at Anyang in 2014–2015, and the experimental protocol was approved by the CRICAAS. The following six traits related to early maturity were investigated in this study: FT, FBP, WGP, NFFB, HNFFB and PH. F_2_ families were investigated by single plant. In addition, for F_2:3_ families, all the field experiments followed a randomized complete block design with three replications. Ten plants in the middle of each F_2:3_ family row were investigated for NFFB, HNFFB and PH. The average was calculated as the last phenotypic value. The phenotypic data were analyzed using R software.

### DNA extraction and SNP genotyping

Genomic DNA was isolated from the two parents and 170 F_2_ individuals using the modified CTAB method [30]. The GBS library was constructed based on the predesigned scheme. Genomic DNA was incubated at 37°C with *Mse* I (New England Biolabs, NEB), T4 DNA ligase (NEB), ATP (NEB), and *MseI* Y adapter N containing a barcode for the F_2_ population. The details of the GBS strategy are described by Zhang et al. [31] and Zhou et al. [32]. The sequences of each sample were sorted according to the barcodes. To ensure that the reads were reliable and without artificial bias (low-quality paired reads, which mainly resulted from base-calling duplicates and adapter contamination) in the subsequent analyses, raw data (raw reads) in fastq format were first processed through a series of quality control (QC) procedures by C scripts. QC standards were as follows: (1) Removing reads with ≥ 10% unidentified nucleotides (N); (2) Removing reads with > 50% bases having phred quality < 5; (3) Removing reads with > 10 nt aligned to the adapter, allowing ≤ 10% mismatches; (4) Removing reads containing *Hae* III or *EcoR* I. BWA (Burrows-Wheeler Aligner) was used to align the clean reads of each sample against the reference genome (settings: mem -t 4 -k 32 –M -R) [33]. Alignment files were converted to BAM files using SAMTools software (settings:–bS–t) [34]. If multiple read pairs had identical external coordinates, only the pair with the highest mapping quality was retained. Variant calling was performed for all samples using the GATK software [35]. SNPs were filtered by the PYTHON script. ANNOVAR, an efficient software tool, was used to annotate SNPs based on the GFF3 files for the *Gossypium hirsutum* genome [36]. Polymorphic markers were classified into eight segregation patterns (ab×cd, ef×eg, hk×hk, lm×ll, nn×np, aa×bb, ab×cc and cc×ab). For the F_2_ population, segregation patterns were chosen for the genetic map. Prior to map construction, the markers with segregation distortion (p < 0.001) or integrity (> 60%) or containing abnormal bases were filtered.

### Genetic map construction

According to the position on the *G*. *hirsutum* genome, the linkage group was divided, and then we used PYTHON script to sort the markers in every linkage group. The linkage map was constructed by JoinMap version 4.0 software using a regression approach with a logarithm of odds (LOD) threshold of 6. We used jump threshold at 0.5 to assign segregating markers to linkage groups [37].

### QTL mapping

A total of 3978 SNPs were used for QTL mapping. The QTL IciMapping software was used to identify QTLs [38]. The composite interval mapping (CIM) method was utilized to detect any significant association between phenotypic traits and marker loci in the datasets. A LOD threshold of 2.5 was used to declare presence of QTLs.

### Candidate gene identification

Candidate genes that were located on chromosome D03 within the 29.51–32.88 Mb QTL region were compared between the parents. To identify candidate genes, all genes in these genomic regions were searched by PYTHON script for gene function in COTTONGENE (https://www.cottongen.org). The genes were functionally annotated using BLASTp [39] and BLAST2GO [40].

### Quantitative real-time PCR

Total RNA was extracted from the samples using the Plant RNA Prep Pure Plant kit (Tiangen, Beijing, China). Then, purified total RNA was reverse-transcribed using the SuperScript III First-Stand Synthesis System to obtain cDNA for qRT-PCR (PrimeScript, Takara, Dalian, China). Real-time PCR was performed on the ABI 7500 Real-Time PCR System (Applied Biosystems, Foster City, CA, USA). The detailed primer information used for PCR amplification is listed in S1 Table. The gene expression levels were calculated using the 2^−ΔΔCT^ method, and each sample used three biological replicates.

## Results

### Phenotypic characteristics of traits related to early maturity in F_2_, F_2:3_ populations and two parents

Phenotypic analysis of the F_2_ and F_2:3_ populations (Table 1) revealed significant variation in all six early-maturity traits. The mean value of each early-maturity trait fell between or outside the values of the two parents. All traits showed transgressive segregation, and the absolute values of skewness and kurtosis for each trait were less than 1, which indicated that all the traits were normally distributed and deemed suitable for QTL mapping. Pearson’s correlation coefficient analysis of the six cotton early-maturity traits in the F_2_ and F_2:3_ populations are presented in Table 2. All the traits measured were positively correlated with each other (Table 2).

**Table 1:** Performance and analysis of early-maturity traits in F_2_, F_2:3_ populations and two parents.

	Parent		F_2:_					F_2:3_				
Traits	P_1_	P_2_	Range	Mean	SD	Kurtosis	Skewness	Range	Mean	SD	Kurtosis	Skewness
	LU28	ZHONG213										
FT (d)	56	42	42.50–62.00	58.74	3.50	0.39	0.31	41.00–62.00	57.61	1.94	-0.32	-0.18
FBP (d)	72	59	37.50–68.50	49.64	5.68	0.78	0.88	39.50–72.50	50.47	4.28	-0.36	0.18
WGP (d)	128	101	96.00–126.00	108.4	7.50	0.59	-0.53	98.00–128.00	108.1	5.19	-0.71	0.13
PH (cm)	81.8	55.75	43.00–80.00	69.65	7.46	0.25	0.03	59.38–87.13	75.78	5.46	-0.16	-0.18
HNFFB (cm)	32.1	12	12.21–31.25	18.71	3.33	0.97	0.94	13.38–25.67	19.28	2.36	0.19	0.14
NFFB	7.1	4.4	4.51–9.52	5.86	0.79	0.32	0.13	4.49–7.49	5.69	0.59	0.08	0.40

**Table 2:** Correlation analysis of six cotton early-maturity traits in F_2_, F_2:3_ populations and two parents.

Traits	FT-F_2_	FBP-F_2_	WGP-F_2_	NFFB-F_2_	HNFFB-F_2_	PH-F_2_
FT-F_2_	1					
FBP-F_2_	0.294**	1				
WGP-F_2_	0.689**	0.894**	1			
NFFB-F_2_	0.305**	0.334**	0.396**	1		
HNFFB-F_2_	0.095	0.181	0.182	0.557**	1	
PH-F2	0.017	0.020	0.066	0.293**	0.425**	1
	FT-F_2:3_	FBP-F_2:3_	WGP-F_2:3_	NFFB-F_2:3_	HNFFB-F_2:3_	PH-F_2:3_
FT-F_2:3_	1					
FBP-F_2:3_	0.296**	1				
WGP-F_2:3_	0.615**	0.935**	1			
NFFB-F_2:3_	0.550**	0.437**	0.565**	1		
HNFFB-F_2:3_	0.496**	0.389**	0.505**	0.561**	1	
PH-F_2:3_	0.022	0.081	0.075	0.212**	0.411**	1

**Indicates the correlation reaches significance at 0.01

### Genotyping by sequencing (GBS)

In this study, GBS libraries were constructed using the Illumina HiSeq 2500 platform on 170 F_2_ populations and their parents. A total of 116.23 GB of data containing 650.41 Mb of reads was obtained; each read contained 150 bp (×2) (S2 Table). Among these data, 93.56% bases were high quality with Q30, and the average guanine cytosine (GC) content was 36.94%. The statistical sequencing depth corresponded to 23.90-fold in the LU28, 16.78-fold in the ZHONG213 and 11.68-fold in the 170 progenies (Table 3). The raw data are archived at the NCBI Sequence Read Archive (SRA) under Accession Number PRJNA381273.

**Table 3:** Summary of sequence data in the parents and F_2_ families.

	Average Q30 (%)		Average GC content (%)	Average depth (×)
LU28		92.58	36.28	23.90
ZHONG213		93.04	35.01	16.78
F_2_		93.56	36.94	11.68

**Fig 2:**
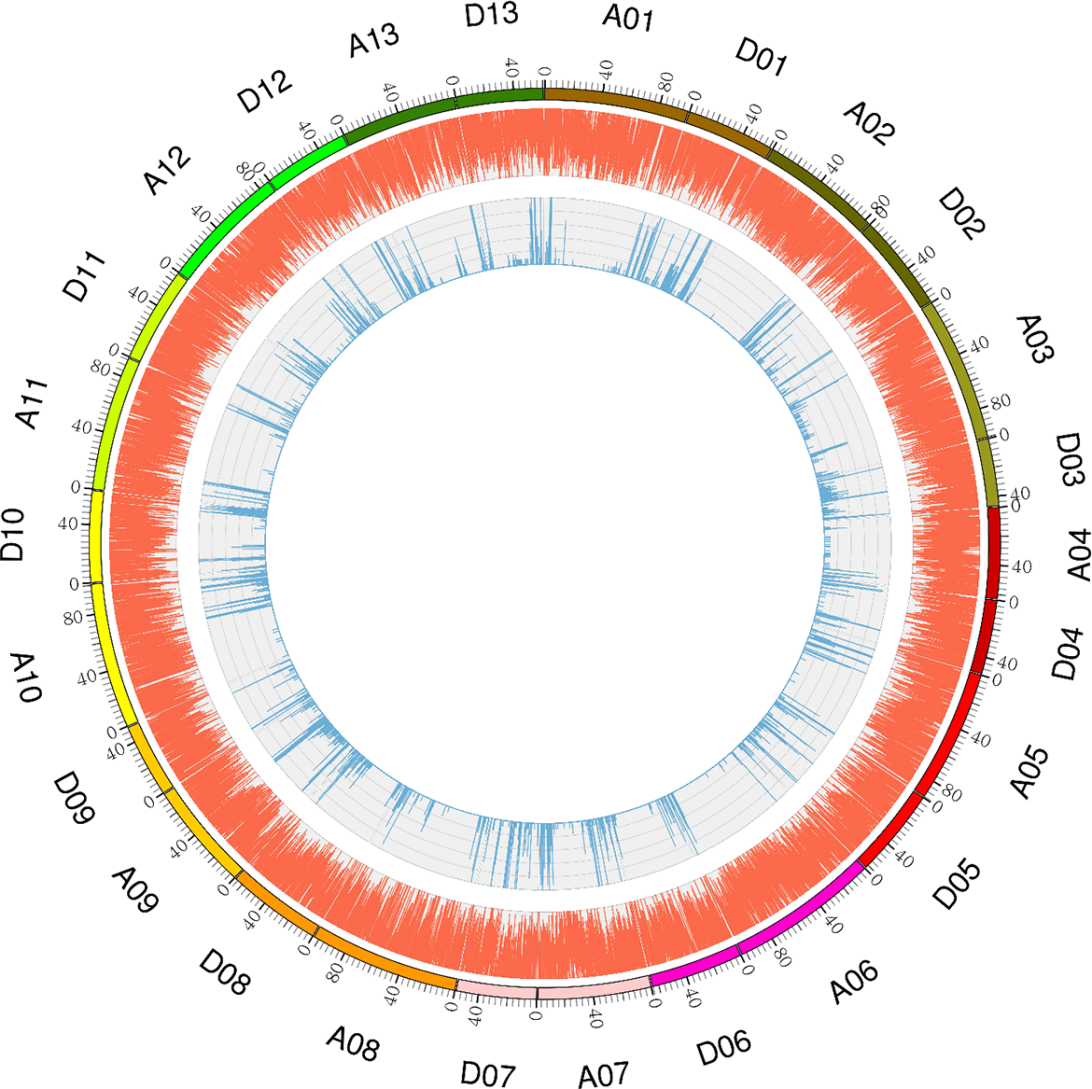
Genome-wide distribution of SNPs throughout the LU28 and ZHONG213 genomes. The outermost box with scale represents the 26 cotton chromosomes. The orange histogram represents the density of SNPs that are polymorphic between LU28 and ZHONG213; the blue histogram indicates the density of aa×bb markers between LU28 and ZHONG213.

A total of 23,576 polymorphic SNP markers were identified from the two parents. All the polymorphic SNP markers contained four genotypes: aa×bb, hk×hk, lm×ll and nn×np (Table 4); however, only the aa×bb genotype was found to be homozygous between the two parents. Therefore, we used this genotype to construct the high-density genetic linkage map. In total, 7,258 SNP markers fell into this type, of which the At and Dt sub-genomes contained 3,645 and 3,613 SNPs, respectively. The percentage of the aa×bb marker on each chromosome varied from 0.85% on chromosome D06 to 9.62% on chromosome A09 (Fig 2).

**Table 4:** Statistics of marker type in the two parents.

Marker Type	Number
nn×np	2,707
aa×bb	7,258
lm×ll	8,625
hk×hk	4,986
Total markers	23,576

### Construction of the genetic map

**Fig 3:**
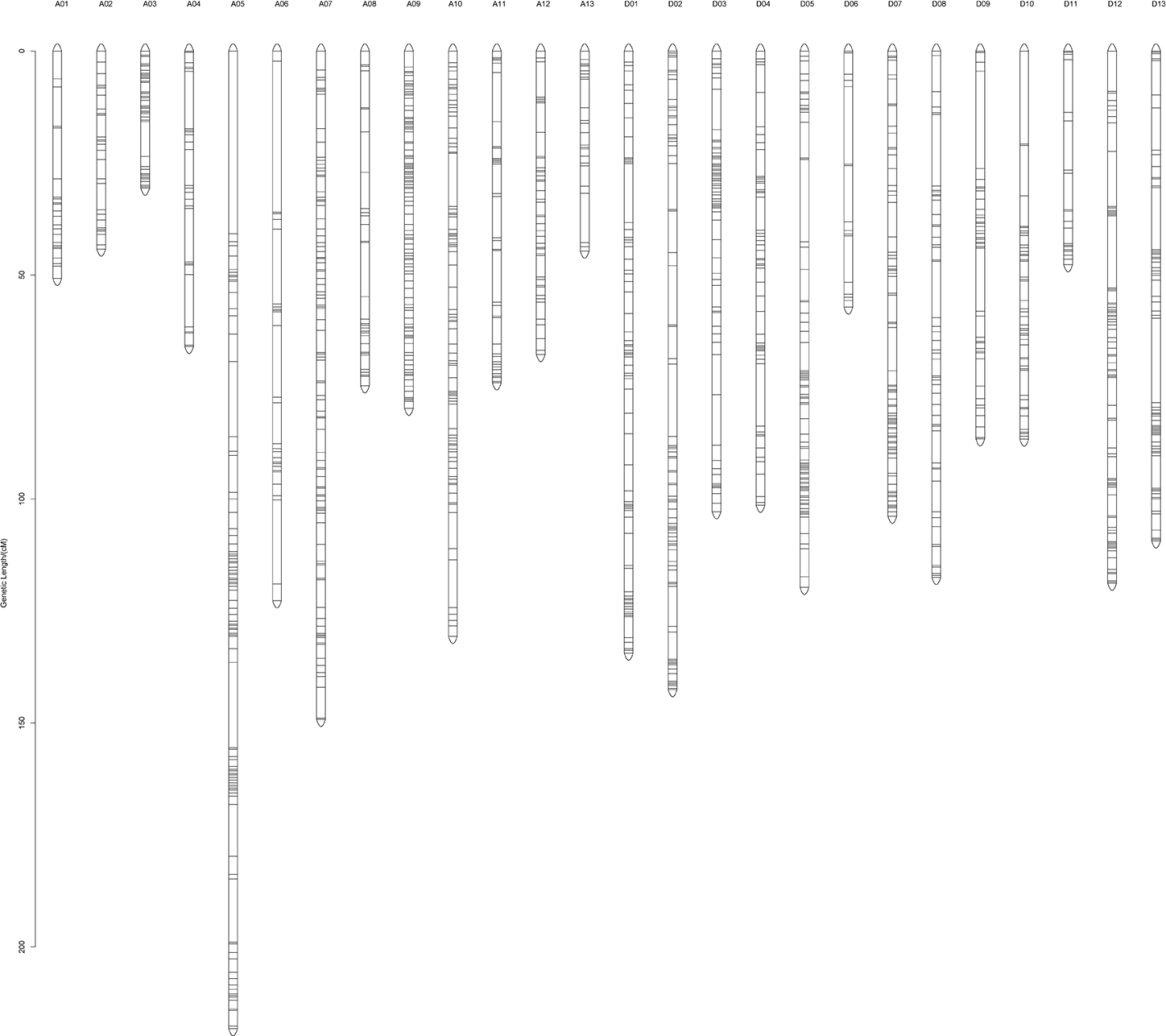
The genetic map constructed by SNP markers.

As mentioned above, we have 23,576 SNPs in all. However, only the type of aa×bb marker could be used in the linkage analysis. After more than 40% missing SNPs were filtered, 3,978 SNP markers were left and mapped on the genetic map. The genetic map of the F_2_ population was distributed over 26 linkage groups (Fig 3, Table 5), which spanned a cumulative distance of 2480 cM, 2,117 loci in the At sub-genome (A01–A13) and 1,861 loci in the Dt sub-genome (D01–D13). Each linkage group ranged from 30.60 cM (A03) to 218.23 cM (A05). The number of SNP markers mapped in each linkage group varied from 34 markers on chromosome D06 to 383 markers on chromosome A09, with an average of 153 SNPs per linkage group (Table 5). The average distance between markers across the 26 linkage groups was 95.39 cM.

**Table 5:** Summary of the high-density SNP Map based on the F_2_ families.

Chromosome	SNP number	Length (cM)	Average interval (cM)	Largest gap (cM)
A01	79	50.75	0.64	12.5
A02	67	44.21	0.66	8.67
A03	145	30.6	0.21	12.44
A04	49	65.95	1.35	16.62
A05	221	218.23	0.99	24.4
A06	117	122.71	1.05	16.85
A07	358	149.19	0.42	10.99
A08	84	74.73	0.89	14.36
A09	383	79.75	0.21	6.45
A10	340	130.66	0.38	15.73
A11	126	74.06	0.59	14.91
A12	94	67.74	0.72	9.67
A13	54	44.66	0.83	10.09
At	2117	1153.24	0.54	24.4
D01	175	142.55	0.81	12.78
D02	185	102.85	0.56	13.19
D03	242	101.4	0.42	18.05
D04	128	119.72	0.94	12.61
D05	147	57.16	0.39	19.03
D06	34	103.85	3.05	13.42
D07	233	117.5	0.5	11.83
D08	81	86.57	1.07	18.66
D09	103	86.63	0.84	18.28
D10	162	47.71	0.29	6.25
D11	48	118.8	2.48	14.22
D12	197	109.37	0.56	14.12
D13	126	133.01	1.06	21.9
Dt	1861	1327.12	0.71	21.9
Total	3978	2480.36	0.62	24.4

At: A sub-genome

Dt: D sub-genome

### Collinearity analysis

**Fig 4:**
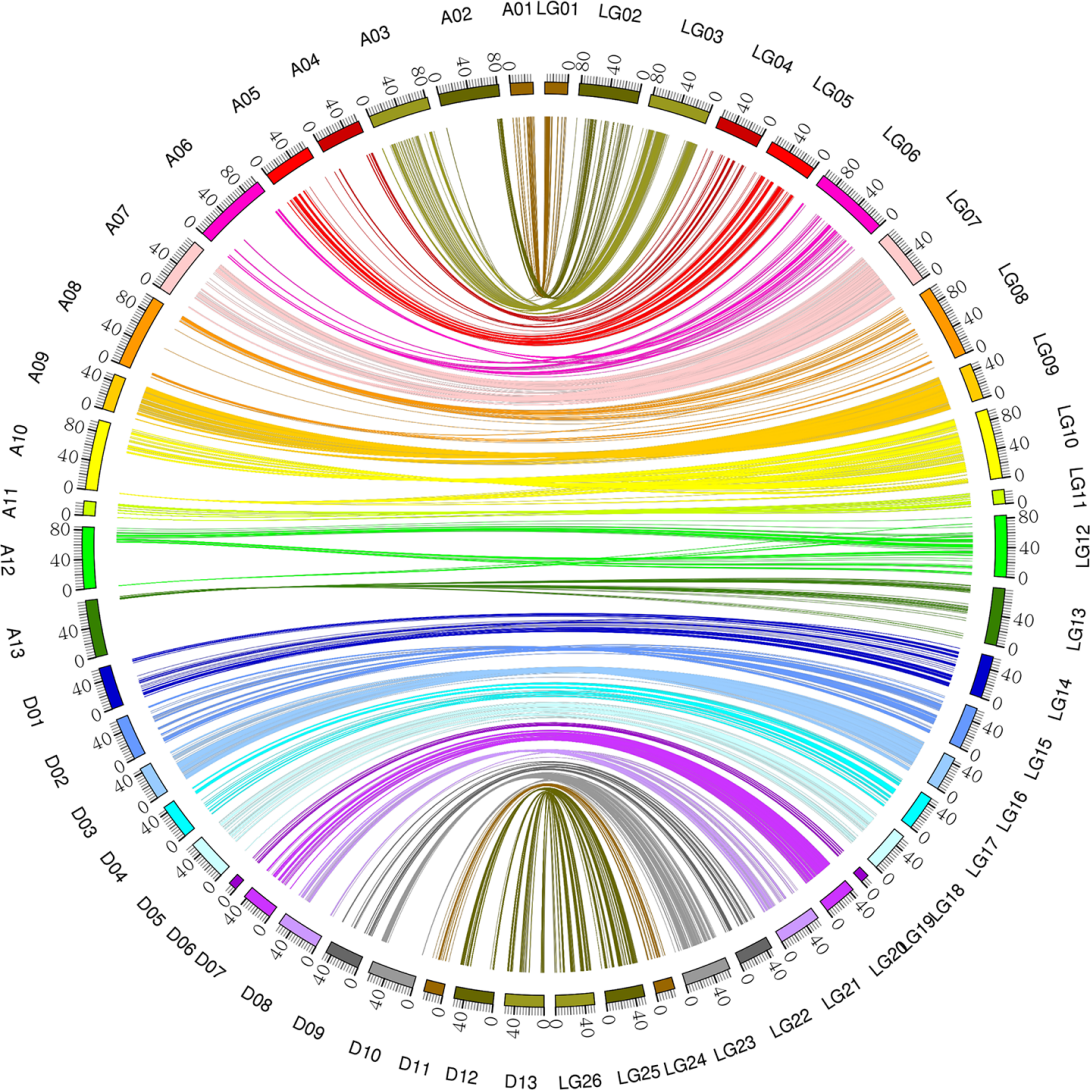
Collinearity between the genetic map (LG01-LG26) and the physical map (A01-A13, D01-D13).

Collinearity analysis between the linkage and physical maps indicated that the linkage map constructed in the present study had good collinearity with the *G*. *hirsutum* reference genome sequence (Fig 4), which suggests the high quality of the F_2_ genetic map. However, several inconsistencies on chromosomes A11, D09 and D10 were also detected. The At and Dt sub-genomes showed good coverage of the reference *G*. *hirsutum* genome, representing 87.37% and 91.02% of the genome assembly length (S3 Table; Fig 4).

### QTL mapping of early-maturity traits in the F_2_ and F_2:3_ families

A total of 47 QTLs for the six early-maturity traits were detected on the 26 chromosomes and explained 2.61–32.57% of the PV. Among the 47 QTLS, there were 12 QTLs for F_2_ generation and 35 QTLs for F_2:3_ generation. Eight QTLs, including two for WGP and NFFB and one each for HNFFB, WGP, FT, PBP and PH, accounted for more than 10% of the PV, whereas the remaining 39 QTLs accounted for less than 10% of the PV (Table 6). Of the total QTLs, only 18 (38%) were located on the At sub-genome, and 29 (62%) were located on the Dt sub-genome. Among these QTLs, 8 (17%) were located on chromosome D03. For FT, the ten QTLs identified were as follows: qFT-A02-1, qFT-A05-1, qFT-A07-1, qFT-A07-2, qFT-A08-1, qFT-A12-1, qFT-A12-2, qFT-D03-1, qFT-D03-2 and qFT-D06-1, which explained 3.57–30.07% of the observed PV, with LOD scores of 2.53–13.80. Ten QTLs for FBP were detected on eight chromosomes. Notably, only 2 (20%) were located on the At sub-genome, whereas 8 (80%) were located on the Dt sub-genome, explaining 3.52–16.96% of the PV. Nine QTLs for WGP were detected on six chromosomes. Here, we note that two significant QTLs, qWGP-D03-1 and qWGP-D03-2, not only had an LOD score > 3 but also explained 30.09% and 10.42% of the PV. A total of five QTLs were detected for PH in this study, and only 1 major QTL explained 10.75% of the PV on chromosome D06 at 20 cM. Four QTLs for HNFFB were identified on the Dt sub-genome and explained 4.53–22.03% of the observed PV, with LOD scores of 2.52–9.44. A total of 9 QTLs were detected for NFFB in this study, of which 2 major QTLs explained 10.88–32.57% of the PV. Three QTLs were on the At sub-genome, and six QTLs were on the Dt sub-genome; these QTLs explained 3.07–32.57% of the observed PV, with LOD scores of 2.52–17.12. Interestingly, a stable QTL region was revealed on chromosome D03 from 43–50 cM and was flanked by FT, FBP, WGP, NFFB and HNFFB. The proportion of PV explained by the QTL ranged from 10.42–32.57%, with LOD scores of 6.66–18.50. Thus, this QTL could be treated as a major QTL for further dissection.

**Table 6:** Stable QTLs for early maturity identified in F_2_ and F_2:3_ populations.

Trait	QTL	Generation	Chromosome	Position (cM)	LOD	R^2^ (%)
FT	qFT-A02-1	F_2:3_	A02	22.00	2.58	3.72
	qFT-A05-1	F_2:3_	A05	179.00	2.53	3.57
	qFT-A07-1	F_2:3_	A07	20.00	2.69	3.99
	qFT-A07-2	F_2_	A07	149.00	2.59	4.21
	qFT-A08-1	F_2:3_	A08	71.00	2.75	4.65
	qFT-A12-1	F_2_	A12	26.00	3.11	5.27
	qFT-A12-2	F_2_	A12	39.00	2.59	4.75
	qFT-D03-1	F_2:3_	D03	0.00	2.61	4.07
	qFT-D03-2	F_2:3_	D03	48.00	13.80	30.07
	qFT-D06-1	F_2:3_	D06	22.00	2.54	4.77
FBP	qFBP-A08-1	F_2:3_	A08	36.00	4.65	8.33
	qFBP-A10-1	F_2:3_	A10	128.00	2.65	4.00
	qFBP-D03-1	F_2:3_	D03	43.00	8.70	16.96
	qFBP-D03-2	F_2:3_	D03	91.00	2.52	3.70
	qFBP-D06-1	F_2:3_	D06	53.00	2.54	3.78
	qFBP-D08-1	F_2:3_	D08	0.00	3.30	5.82
	qFBP-D10-1	F_2:3_	D10	78.00	2.55	4.16
	qFBP-D10-2	F_2_	D10	2.00	2.66	6.93
	qFBP-D12-1	F_2:3_	D12	83.00	2.57	3.52
	qFBP-D13-1	F_2_	D13	88.00	2.55	5.52
WGP	qWGP-A08-1	F_2:3_	A08	0.00	2.60	2.61
	qWGP-A08-2	F_2:3_	A08	36.00	5.89	7.79
	qWGP-A12-1	F_2:3_	A12	34.00	3.54	4.60
	qWGP-A12-2	F_2_	A12	45.00	2.60	2.91
	qWGP-D01-1	F_2:3_	D01	81.00	3.04	3.90
	qWGP-D03-1	F_2:3_	D03	49.00	18.50	30.09
	qWGP-D03-2	F_2_	D03	50.00	6.66	10.42
	qWGP-D08-1	F_2:3_	D08	0.00	5.43	7.16
	qWGP-D10-1	F_2:3_	D10	74.00	4.07	5.40
PH	qPH-A01-1	F_2:3_	A10	15.00	2.73	5.98
	qPH-A08-1	F_2_	A08	35.00	3.25	4.92
	qPH-D08-1	F_2_	D08	78.00	3.53	5.67
	qPH-D06-1	F_2:3_	D06	20.00	3.48	10.75
	qPH-D08-1	F_2:3_	D08	111.00	2.57	5.18
HNFFB	qHNFFB-D03-1	F_2:3_	D03	48.00	9.44	22.03
	qHNFFB-D06-1	F_2:3_	D06	25.00	3.90	8.31
	qHNFFB-D09-2	F_2_	D09	29.00	2.52	6.06
	qHNFFB-D12-1	F_2:3_	D12	34.00	2.52	4.53
NFFB	qNFFB-A05-1	F_2:3_	A05	46.00	2.54	3.11
	qNFFB-A07-1	F_2:3_	A07	128.00	2.90	4.45
	qNFFB-A12-1	F_2:3_	A12	1.00	6.66	10.88
	qNFFB-D02-1	F_2:3_	D02	70.00	2.69	3.87
	qNFFB-D03-1	F_2:3_	D03	49.00	17.12	32.57
	qNFFB-D05-1	F_2_	D05	104.00	2.53	3.91
	qNFFB-D06-1	F_2:3_	D06	56.00	2.52	3.07
	qNFFB-D10-1	F_2:3_	D10	49.00	2.60	3.60
	qNFFB-D13-1	F_2_	D13	109.00	2.62	4.57

### Identification of a candidate gene for traits related to early maturity on chromosome D03

**Fig 5:**
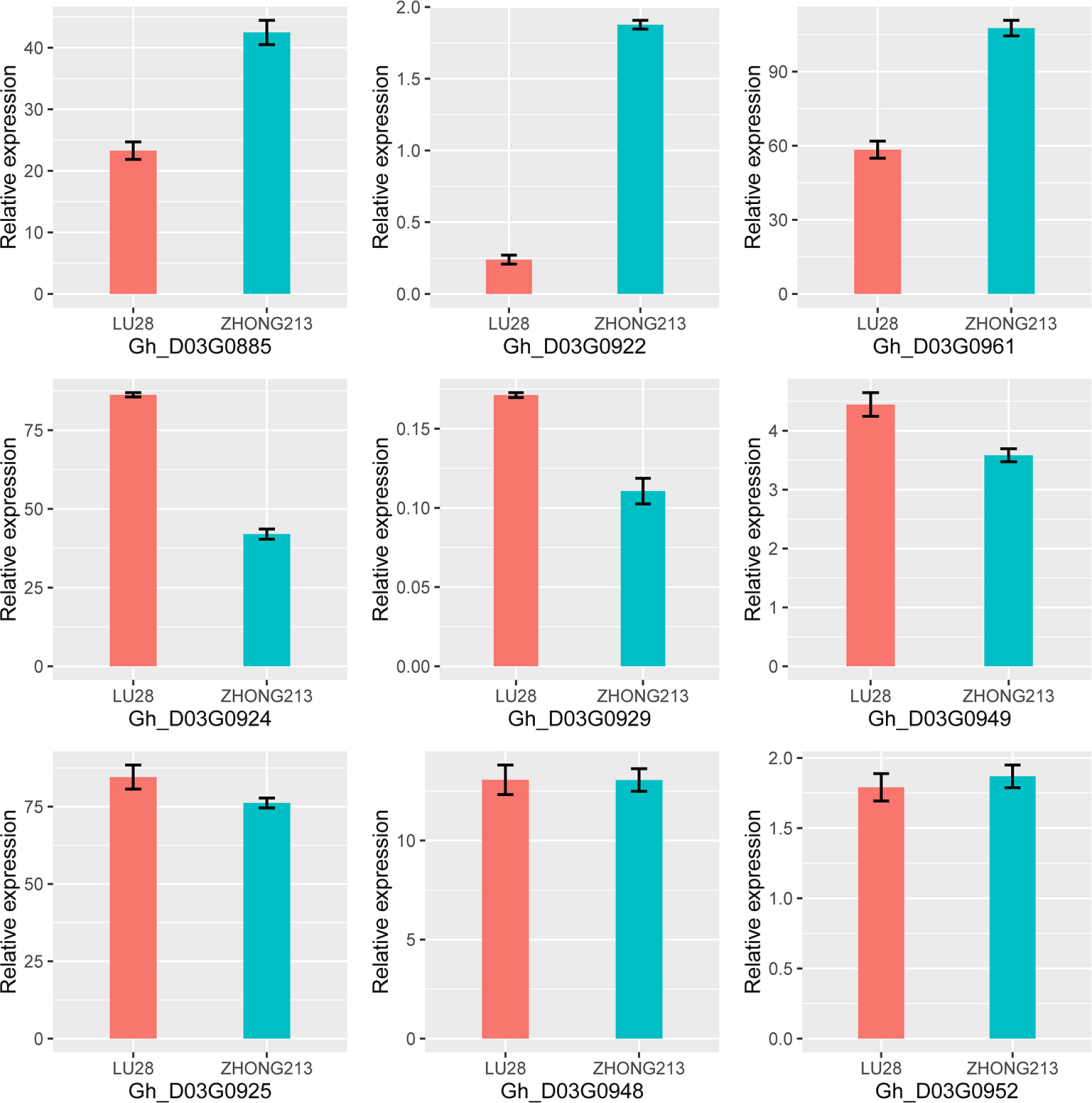
Expression of the genes in the two-leaf stage determined by qRT-PCR.

As mentioned above, qFT-D03-2, qFBP-D03-1, qWGP-D03-1, qWGP-D03-2 qHNFFB-D03-1 and qNFFB-D03-1 were the six steady QTLs that showed significant effects on FT, FBP, WGP, HNFFB and NFFB from 43–50 cM (Table 6) and occupied a physical region of 3.36 Mb on chromosome D03. Based on the comparative mapping of the *G*. *hirsutum* reference genome [9], 112 genes were predicted (S4 Table). Among these genes, 14 candidate genes had no annotation information. Data analysis of qRT-PCR revealed that the expression levels of three genes, *Gh_D03G0885*, *Gh_D03G0922* and *Gh_D03G0961*, were significantly lower in LU28 than in ZHONG213, whereas the expression levels of *Gh_D03G0924*, *Gh_D03G0929* and *Gh_D03G0949* were higher in LU28 than in ZHONG213. The expression levels of the remaining genes in LU28 were not significantly different from those of the genes in ZHONG213 (Fig 5).

## Discussion

With the progress or completion of genome sequencing of many important crops, the molecular genetics and breeding of crops have entered into a high-throughput, large-scale whole-genome level molecular design platform. SNP molecular markers have gained great attention in molecular marker-assisted breeding programs, population and evolutionary genetics, bi-parental QTL mapping, and association mapping studies [10, 11, 41] because they are widely distributed, highly polymorphic and large in quantity [42–44].

The characteristics of markers determine the distribution density of a marker on a genetic linkage map, which further affects the accuracy of the QTLs. From the first-generation marker to the third-generation marker, the distribution of markers in the whole genome of cotton is becoming increasingly abundant, which greatly improves the density of the marker. In this study, we employed GBS to identify a major QTL related to early maturity in cotton using an F_2_ mapping population. This technique is rapid, cost-effective and widely used in many crops [14, 45–48]. Our map contained 3978 polymorphic SNP markers, and the average distance between markers was 0.62 cM. Compared with previous studies, regardless of whether RFLP, SSR or SNP markers constructed traditional linkage maps generated from the same cross, the total length of the map in our study is shorter [10, 36, 49, 50]. The main reasons for these differences are caused by the marker types, the size of the population and the type of mapping population. Moreover, in our 26 chromosomes, the distribution of some chromosome markers is nonuniform and the number of markers is low, which can decrease the genetic distance of genetic map. The shortest A03 chromosome, although having 145 markers, is unevenly distributed on chromosomes. The three short chromosomes (chromosomes A01 A02 and A13) only spanned 50.75 cM, 44.21 cM and 44.66 cM, harboring 79, 54 and 67 markers, respectively. However, the marker density of cotton has obviously improved, which is mainly attributed to the development and application of high-throughput sequencing. Wang et al. used 4,999,048 SNPs to construct an ultra-dense genetic map extended over 4,042 cM [50]. This map provides a meaningful reference for other scientists who are engaged in *G*. *hirsutum* genome assembly [9]. By taking advantage of the RAD-seq technique in upland cotton, the construction of more comprehensive genetic maps using SSR and RAD is becoming increasingly popular [10, 51]. Hulse-Kemp et al. constructed a genetic map that had 19,191 SNPs and 0.21-cM distribution density [52]. Zhang et al. used SALF-seq to construct a high-density genetic map comprised of 5,521 SNPs with 0.78-cM marker spacing [16]. These studies demonstrate that the number of high-throughput sequencing techniques is rapidly growing. The number of markers employed in this study is much higher than that in previous studies and greatly improved the marker density of the linkage map [10, 16]. Moreover, in our study, the number of large interval segments of the map is much lower than that of previous studies [16, 36, 49, 53, 54], which suggests the higher accuracy of the detected QTLs and the possibility of detecting large QTL interval regions in other molecular markers.

Collinearity is an important factor in determining the quality of a genetic map. Collinearity analysis showed that the constructed map had good collinearity with the *G*. *hirsutum* reference genome [9], which indicates the high quality and accuracy of the map. The coverage of genomes by the At sub-genome and Dt sub-genome is 87.37% and 91.02%, respectively. One main reason for the lower coverage by the At sub-genome than the Dt sub-genome is that two linkage groups, A03 and A13, represent only 70.27% and 78.80% of the corresponding chromosomes, respectively, while others chromosome range from 83.23% to 98.49% (S4 Table).

Early maturity is an important cotton trait, and short-season cotton is an important cotton resource in China. Given the need to ensure food security, it is necessary to increase the production level and improve yield and quality in cotton-growing regions. Early maturity of cotton is a comprehensive trait that includes WGP, FT, NFFB and other traits, which are important indicators of earliness [3, 4, 10]. These traits are quantitative traits controlled by multiple genes [55, 56].

In cotton, a few studies have mapped genomic regions associated with early-maturity traits. These studies included intra-specific crosses of *G*. *hirsutum* TM1 × CRI36 [5], Baimian2 × TM-1 [4], Baimian2 × CRI12 [4] and CRI36 × G2005 [10]. As shown in the pedigree (S1 Fig), ZHONG213, CRI36 and Baimian2 were all derived from the King, which is the founder of the most of China’s short-season cultivars. CRI36 and Baimian2 is a short-season cotton bred in 1999 and 2008, and the WGP was approximately 115 and 110 days respectively. However, with the improvement of breeding technology, ZHONG213 as a new line, with a WGP of 105 days; compared to CRI36 and Baimian2, it has a shorter WGP, better quality, good disease resistance and wide adaptability. It is worth mentioning that none of the parents involved in these studies were insect resistant. Thus, the accuracy of FT and WGP explained in these studies might be affected by insect infestation, such as cotton bollworm, which feeds on buds. To overcome this limitation and ensure the accuracy of the phenotypic data investigated in our study, we used BT cotton parents that have high resistance to the field environment.

**Fig 6:**
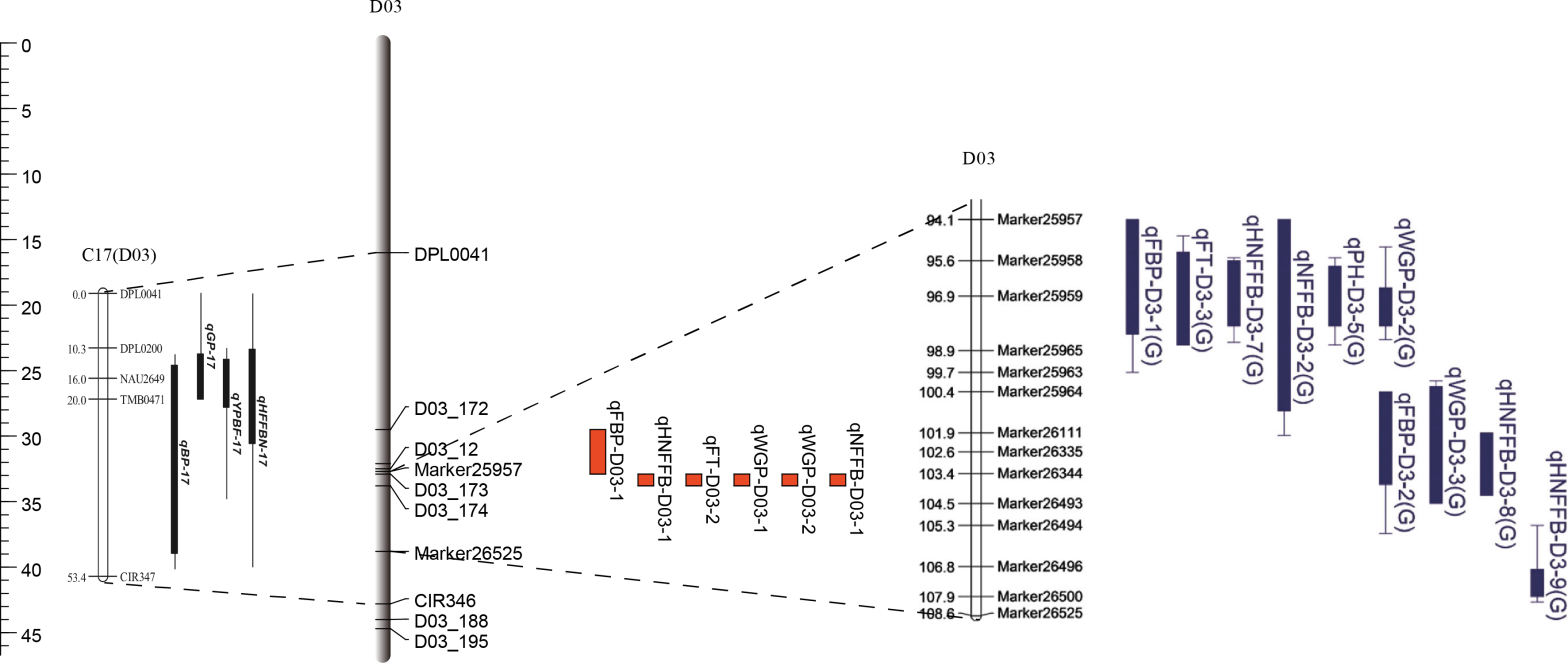
Physical maps and linkage relationships among quantitative trait loci (QTLs) in previous and present studies.

In the present study, we uncovered a total of 47 QTLs, including ten for FBP, five for PH, four for HNFFB, ten for FT, seven for WGP, and nine for NFFB (Table 6). Most importantly, the main QTLs for FT (qFT-D03-2), FBP (qFBP-D03-1), WGP (qWGP-D03-1/ qWGP-D03-2), HNFFB (qHNFFB-D03-1) and NFFB (qNFFB-D03-1) were overlapped on chromosome D03 from 43–50 cM and explained 10.42–32.57% of the PV explained (PVE). In particular, one common QTL qWGP-D03-1/ qWGP-D03-2 for WGP was detected in both generations. Common QTLs should be reliable and could be used in maker-assisted selection to increase cotton early maturity. From previous studies, early-maturity QTLs were detected on almost all 26 chromosomes. However, in recent years, many researchers have demonstrated that chromosome D03 contains important segments for early-maturity traits, which indicates that chromosome D03 is responsible for early maturity [3, 4, 10]. When comparing our results with those previously reported by studies of early maturity, the main QTLs on the D03 chromosome could be validated based on previous linkage mapping. The six steady QTLs were positioned between DPL0041 and CIR347 [4] and had an overlap with five early-maturity QTLs that have been reported between Marker25958 and Marker25963 on chromosome D03 [10] (Fig 6). Subsequently, Su et al. found that these SSR markers mapped to the genome sequence by electronic PCR (e-PCR) and, using association mapping, found one peak SNP locus mapped to D03 at 31.98 Mb, positioned between DPL0200 and CIR347 [3]. Interestingly, we also discovered the same SNP located across the same markers and further narrowed the candidate region to 29.51–32.88 Mb on chromosome D03. These findings validate the QTL results and increase cotton breeders’ confidence in the identity of the main QTL.

One hundred and twelve genes were predicted and annotated in this interval. Among them, *Gh_D03G0885* and *Gh_D03G0922* caught our attention because their expression levels were higher in the early-maturity variety ZHONG213 than in the late-maturity variety LU28. In particular, *Gh_D03G0922* was the only *AGL8* homolog in the cotton genome, and *AGL8* was the best match to Gh_D03G0922 in the *Arabidopsis* genome. *AGL8* is a member of MADS-box gene family, which plays significant roles in regulating FT and flower initiation and participates in plant growth and development [57, 58]. Our previous association mapping study also showed that *Gh_D03G0922* is a potential candidate gene for early maturity [3]. Therefore, we speculate that *Gh_D03G0922* plays a major role in cotton flowering similar to *AGL8* in *Arabidopsis*. The results of BLAST alignment showed that the CDS identity of *Gh_D03G0885* with the *Arabidopsis TOC1* gene was as high as 58%. Furthermore, *Gh_D03G0885* encoded a protein sharing 49% sequence identity with the *Arabidopsis* TOC1 protein. Hence, we regard *Gh_D03G0885* as the *TOC1* homolog in cotton. *TOC1* contributes to plant fitness by influencing the circadian clock period, and the expression of *TOC1* is correlated with rhythmic changes in chromatin organization [59, 60]. Therefore, it is reasonable to postulate that *Gh_D03G0885* and *Gh_D03G0922* are candidate genes for early maturity in cotton. Of the remaining 110 genes in this region, 14 annotated genes were found to be unknown genes, and of the remaining 94 genes, none were identified as homologous to *Arabidopsis* genes that are involved in the FT pathway, such as *CO*, *SOC1*, *FD*, and *TFL1*. Therefore, to prove the hypothesis, further experiments are needed.

## Supporting information

S1 Fig The pedigree figure of cultivars ZHONG213, CRI36 and Baimian2. (TIF)

S1 Table qRT-PCR primers. (XLS)

S2 Table Sequencing statistics for ZHONG213, LU28 and 170 F2 families. (XLS)

S3 Table The collinearity results of genetic map and physical map. (XLS)

S4 Table Summary of 112 genes’ annotation information. (XLS)
