# Supplementary figures and images for: High-density genetic linkage map construction by F2 populations and QTL analysis of early-maturity traits in upland cotton (Gossypium hirsutum L.)
Source: PLoS One. 2017 Aug 15;12(8):e0182918. doi: 10.1371/journal.pone.0182918 (PMC5557542; doi:10.1371/journal.pone.0182918)

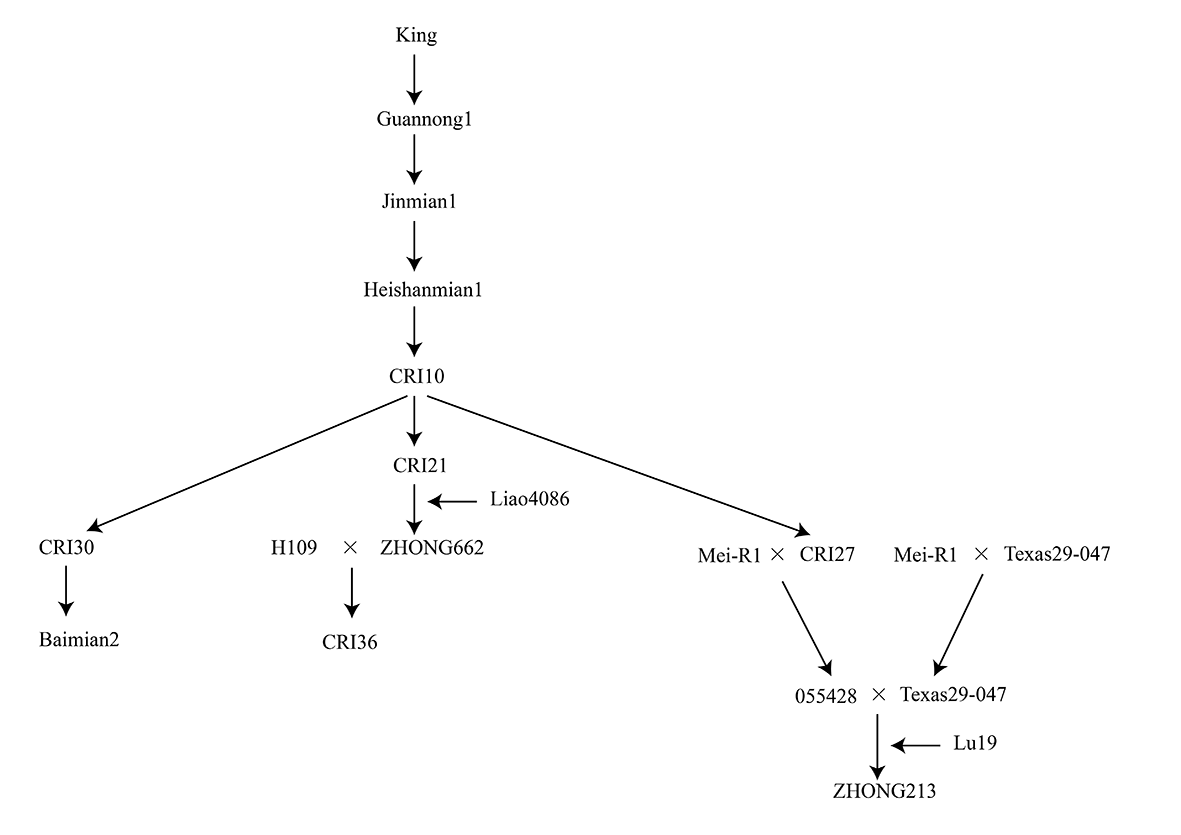

Supplement: S1 Fig — (TIF) [file pone.0182918.s001.tif]
